# A core-shell structured COVID-19 mRNA vaccine with favorable biodistribution pattern and promising immunity

**DOI:** 10.1038/s41392-021-00634-z

**Published:** 2021-05-31

**Authors:** Ren Yang, Yao Deng, Baoying Huang, Lei Huang, Ang Lin, Yuhua Li, Wenling Wang, Jingjing Liu, Shuaiyao Lu, Zhenzhen Zhan, Yufei Wang, Ruhan A, Wen Wang, Peihua Niu, Li Zhao, Shiqiang Li, Xiaopin Ma, Luyao Zhang, Yujian Zhang, Weiguo Yao, Xingjie Liang, Jincun Zhao, Zhongmin Liu, Xiaozhong Peng, Hangwen Li, Wenjie Tan

**Affiliations:** 1 NHC Key Laboratory of Biosafety, National Institute for Viral Disease Control and Prevention, Chinese Center for Disease Control and Prevention, Beijing, China; 2 Stemirna Therapeutics, Shanghai, China; 3 National Institute for Food and Drug Control, Beijing, China; 4 National Kunming High-level Primate Research Center, Institute of Medical Biology, Chinese Academy of Medical Sciences and Peking Union Medical College, Yunnan, China; 5 Shanghai East Hospital, Tongji University, Shanghai, China; 6 CAS Center for Excellence in Nanoscience, CAS Key Laboratory for Biomedical Effects of Nanomaterials and Nanosafety, Chinese Academy of Sciences and National Center for Nanoscience and Technology of China, Beijing, China; 7 University of Chinese Academy of Sciences, Beijing, China; 8 State Key Laboratory of Respiratory Disease, National Clinical Research Center for Respiratory Disease, Guangzhou Institute of Respiratory Health, The First Affiliated Hospital of Guangzhou Medical University, Guangzhou, Guangdong, China; 9 Center for Biosafety Mega-Science, Chinese Academy of Sciences, Wuhan, Hubei China

**Keywords:** Translational research, Vaccines

## Abstract

Although inoculation of COVID-19 vaccines has rolled out globally, there is still a critical need for safe and effective vaccines to ensure fair and equitable supply for all countries. Here, we report on the development of a highly efficacious mRNA vaccine, SW0123 that is composed of sequence-modified mRNA encoding the full-length SARS-CoV-2 Spike protein packaged in core–shell structured lipopolyplex (LPP) nanoparticles. SW0123 is easy to produce using a large-scale microfluidics-based apparatus. The unique core–shell structured nanoparticle facilitates vaccine uptake and demonstrates a high colloidal stability, and a desirable biodistribution pattern with low liver targeting effect upon intramuscular administration. Extensive evaluations in mice and nonhuman primates revealed strong immunogenicity of SW0123, represented by induction of Th1-polarized T cell responses and high levels of antibodies that were capable of neutralizing not only the wild-type SARS-CoV-2, but also a panel of variants including D614G and N501Y variants. In addition, SW0123 conferred effective protection in both mice and non-human primates upon SARS-CoV-2 challenge. Taken together, SW0123 is a promising vaccine candidate that holds prospects for further evaluation in humans.

## Introduction

Since the outbreak of COVID-19, a large number of vaccine programs have been initiated using a variety of SARS-CoV-2 antigen and vaccine modalities such as live-attenuated virus, inactivated virus, viral protein-expressing adenovirus, recombinant viral protein, and nucleic acid (DNA and mRNA) vaccines encoding either full-length or partial viral antigen.^1^ Some of them have demonstrated protective capacity in animal models and promising immunogenicity in humans.^2–7^ However, it remains to be demonstrated which type of vaccine will eventually provide the most efficient protection without causing adverse effects in humans. In addition, it is becoming increasingly evident that an effective vaccine needs to elicit durable responses due to rapid waning immunity observed in COVID-19 patients,^8,9^ and in the meantime provides protection against emerging SARS-CoV-2 variants with increased infectivity and transmissibility such as the D614G variant.^10^ A fundamental understanding of the immune mechanisms associated with broad protection is therefore critical to guide the ongoing COVID-19 vaccine development. In this regard, vaccines that are able to mount both antibody (Ab) and virus-specific T cell responses will likely be most effective in protecting against multiple virus mutants.^11,12^ Nucleic acid vaccines including DNA and mRNA vaccines have not only shown high immunogenicity in eliciting both humoral and T cell responses, but also offered low cost on production with a short development and manufacturing timeline.^13^ Consequently, a number of COVID-19 mRNA candidate vaccines had been successfully developed shortly after the genomic sequence of SARS-CoV-2 was identified.^5,7^ Some of these vaccines were the first to enter clinical trials and have been licensed by FDA.^14^

Since nucleic acid-based antigen sources cannot enter the antigen-presenting cells (APCs) directly, they are usually delivered to cells either through electroporation or in certain types of particulate forms. Packaging mRNA molecules in nanoparticles also have the benefit of avoiding degradation by RNases and ensuring effective APCs targeting and antigen production, which are prerequisites for induction of vaccine responses.^13,15^ While a variety of delivery systems for mRNA vaccine have been developed, there are still concerns regarding unfavorable biodistribution pattern due to their unique size and composition, and issues associated with their production, stability and storage conditions.^16–18^ In an effort to circumvent these issues, we applied a core–shell structured lipopolyplex (LPP) platform for mRNA vaccine production. In this platform, mRNA molecules encoding the protein antigens bind tightly to a positively charged cationic compound (SW-01) to form a dense core structure that is encapsulated in a lipid shell. The platform shares a number of features with lipid nanoparticles (LNP) that have been extensively used for siRNA delivery and adopted for mRNA vaccine development.^18,19^ Since the LPP particle resembles the overall structure of a virus, it provides certain advantages such as facilitated cell uptake and stimulation of key signal transduction pathways that are essential for activation and maturation of APCs, such as Toll-like receptor 7/8 signaling. In this study, LPP also showed high transfection efficiency and sustained expression of targeted mRNA molecules in APCs such as DC2.4 cells. Besides, in comparison with traditional LNP, LPP expressed mRNA mostly in injection site after intramuscular inoculation and preferred expressed mRNA in spleen instead of liver after its leakiness in blood vessel, which may alleviate the worry about the potential off target effect and systemic toxicity.

In this study, we prepared an LPP-based mRNA vaccine (SW0123) against SARS-CoV-2. A complex of mRNA encoding the full-length SARS-CoV-2 Spike (S) and SW-01 served as the core, and a mixture of ionized and non-ionized lipids formed the shell. The core–shell structured vaccine particles were prepared with a microfluidics-based apparatus which is compatible with large-scale production. We rigorously characterized the vaccine particles to ensure high quality, reproducibility and long-term stability. Evaluation in mouse and nonhuman primate models showed that SW0123 was highly immunogenic in eliciting both antibody and T cell responses, and more importantly was able to confer robust protection against SARS-CoV-2 challenge. Moreover, antibodies elicited by SW0123 demonstrated high neutralizing potency against a panel of SARS-CoV-2 variants including the D614G and N501Y mutant that has emerged as the dominant variant with increased infectivity.^20–22^ Lastly, intramuscular administration of SW0123 demonstrated a favorable biodistribution pattern with limited accumulation in the liver or other major organs, thus indicating a benign and desirable toxicity profile. SW0123 is currently being evaluated in a Phase I clinical trial in China.

## Results

### SW0123 has favorable in vitro and in vivo characteristics as a prophylactic vaccine

**Fig. 1:**
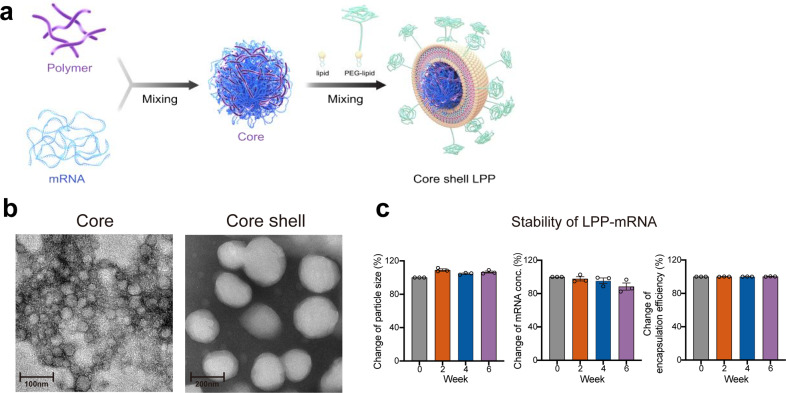
Preparation and characterization of a “core–shell”-structured LPP-mRNA vaccine. **a** Schematic view on preparation of SW0123 mRNA vaccine. **b** Representative transmission electronic microscopy (TEM) image of SW0123 vaccine particles. Left: mRNA/SW-01 complexes. Right: LPP particles with a complex lipid cover. **c** Time-dependent changes in particle size, mRNA concentration and encapsulation efficiency of SW0123 upon storage at 4 °C

**Fig. 2:**
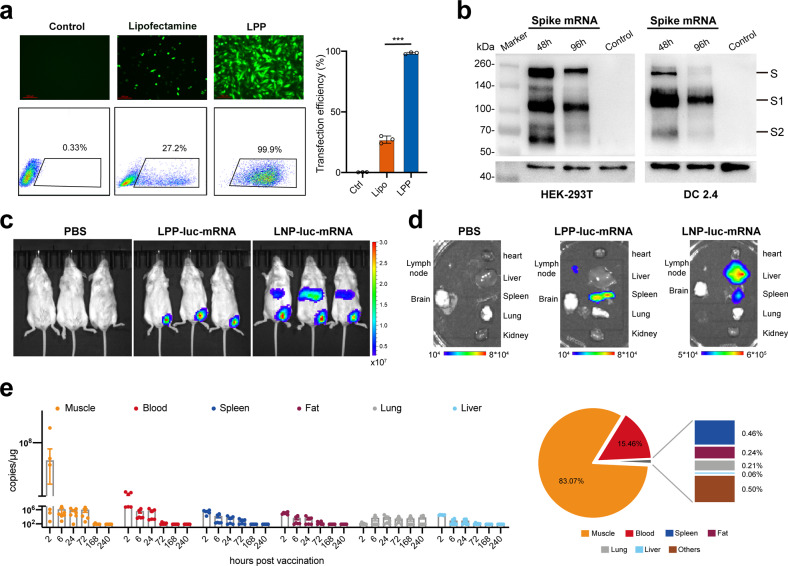
Transfection efficiency and biodistribution of LPP-mRNA. **a** eGFP-mRNA was transfected into DC2.4 cells using LPP or lipofectamine. Cells were harvested after 24 h of incubation. eGFP expression was visualized using fluorescent microscope (upper panel) and quantified using flow cytometry (lower panel). **b** S protein expression from mRNA used for SW0123 preparation. mRNA encoding the S protein was transfected into HEK-293T and DC2.4 immortalized cells. Cells were harvested 48 and 96 h later, and S protein levels in cell lysate were detected with western blot. **c** Comparison of biodistribution between LPP/mRNA and LNP/mRNA. BALB/c mice were administrated intramuscularly (i.m.) with LPP or LNP encapsulated with luciferase-mRNA. Bioluminescence was measured 6 h later in a Xenogen IVIS-200 imaging system. **d** Mice were euthanized, and bioluminescent intensity in major organs were detected with a Xenogen IVIS-200 imaging system. **e** Biodistribution of SW0123 in BALB/c mice. BALB/c mice were i.m. injected with SW0123 at a single dose of 1.5 mg/kg. Major organs or tissues were collected at different time points following vaccination (*n* = 6 mice each time point). mRNA concentration was determined with real-time qPCR using probes specific for the mRNA component of SW0123 (left panel). Area under curve (AUC) representing accumulative distribution of SW0123 in each specimen is shown (right panel)

A two-step microfluidics-based procedure was applied to prepare LPP-mRNA vaccine particles (Fig. 1a). Transmission electron microscopy (TEM) confirmed the nanoparticle structure with a well-defined dense core at 45 nm diameter (Fig. 1b, left). After microfluidics-based procedure, a layer of lipid shell was surrounded to dense core adding the particle size to 200 nm diameter (Fig. 1b, right). LPP-mRNA vaccine was further characterized for stability by monitoring several parameters upon storage at 4 °C for up to 6 weeks. Particle size, concentration of encapsulated mRNA cargo, and encapsulation efficiency all remained consistent over a 6-week duration, which indicated a high colloidal stability of LPP delivery system (Fig. 1c). To characterize the transfection ability of LPP-mRNA, mRNA encoding enhanced green fluorescent protein (eGFP) was applied as a model molecule, we detected eGFP expression in 99% of immortalized DC2.4 cells following incubation with LPP-eGFP-mRNA. The expression efficiency was almost four times as high as that in the same cell line transfected with eGFP-mRNA using the widely used commercial lipofectamine reagent (Fig. 2a). Using this system, a COVID-19 mRNA vaccine named SW0123 was prepared using sequence-optimized mRNA encoding the full-length S protein as the immunogen. The mRNA molecule was tested in multiple cell lines, and stable and efficient expression of S protein following transfection was confirmed (Fig. 2b). Translation efficiency was further evaluated in vivo using mRNA encoding luciferase as a model molecule. LPP-luciferase-mRNA stored at 4 °C for up to 8 weeks was i.m. administrated into mice, and a consistent and stable signal was detected at injection sites (muscle) (Supplementary Fig. 1).

Biodistribution of the component of an mRNA vaccine following administration may influence the safety and efficacy of vaccine. Next, LPP formulated with mRNA encoding luciferase as a model molecule was evaluated for biodistribution pattern and was compared side-by-side with mRNA-luciferase packaged in LNP. At 6 h of post-intramuscular (i.m.) injection into hind legs, robust luciferase expression at the injection site (muscle) was detected in both groups (Fig. 2c). In the LNP-mRNA group, a strong bioluminescent signal was observed in the liver, which in contrast was absent in the mice receiving LPP-mRNA treatment (Fig. 2c). Further ex vivo dissection showed a robust accumulation of LPP-mRNA in the spleens and in the lymph nodes draining the injection sites, which are major lymphoid organs where vaccine-specific adaptive responses would be initiated (Fig. 2d). While in the LNP-mRNA group, signals were largely detected in the liver, which was in accordance with previous report.^23^ These different biodistribution patterns between LPP-mRNA and LNP-mRNA were most likely, due to their different particle size and lipid composition.

Next, we investigated the kinetic biodistribution of SW0123. Following a single dose (1.5 mg/kg) i.m. administration into hind leg, multiple organs, or tissues were dissected and analyzed with quantitative PCR to quantify the mRNA component of SW0123. Bioluminescent signals were dominantly restricted to the injection sites with the highest signals detected at 2 h of post-injection (Fig. 2e, left). The rest of inoculated SW0123 was mainly distributed in blood circulation. Only a minor proportion was detected in the liver, which was in line with the aforementioned observation using mRNA encoding luciferase as a model molecule. Accumulative distribution of mRNA over a 10-day duration was also determined, and 83% of the inoculated dose of SW0123 was retained at the injection site (muscle). A proportion (15.5%) of SW0123 was found in the blood, and the remaining minority were distributed to multiple tissues including spleen (0.46%), fat (0.24%), and lung (0.21%). Only 0.06% was detected in the liver, reconfirming the unique biodistribution pattern of LPP-mRNA vaccine (Fig. 2e, right). Overall, these data indicate that the LPP-mRNA platform has a favorable biodistribution profile devoid of liver accumulation, and in the meantime can maintain efficient and sustained expression of the mRNA cargo.

### SW0123 induced sustained Ab responses with broadly neutralizing activities against a panel of SARS-CoV-2 variants

**Fig. 3:**
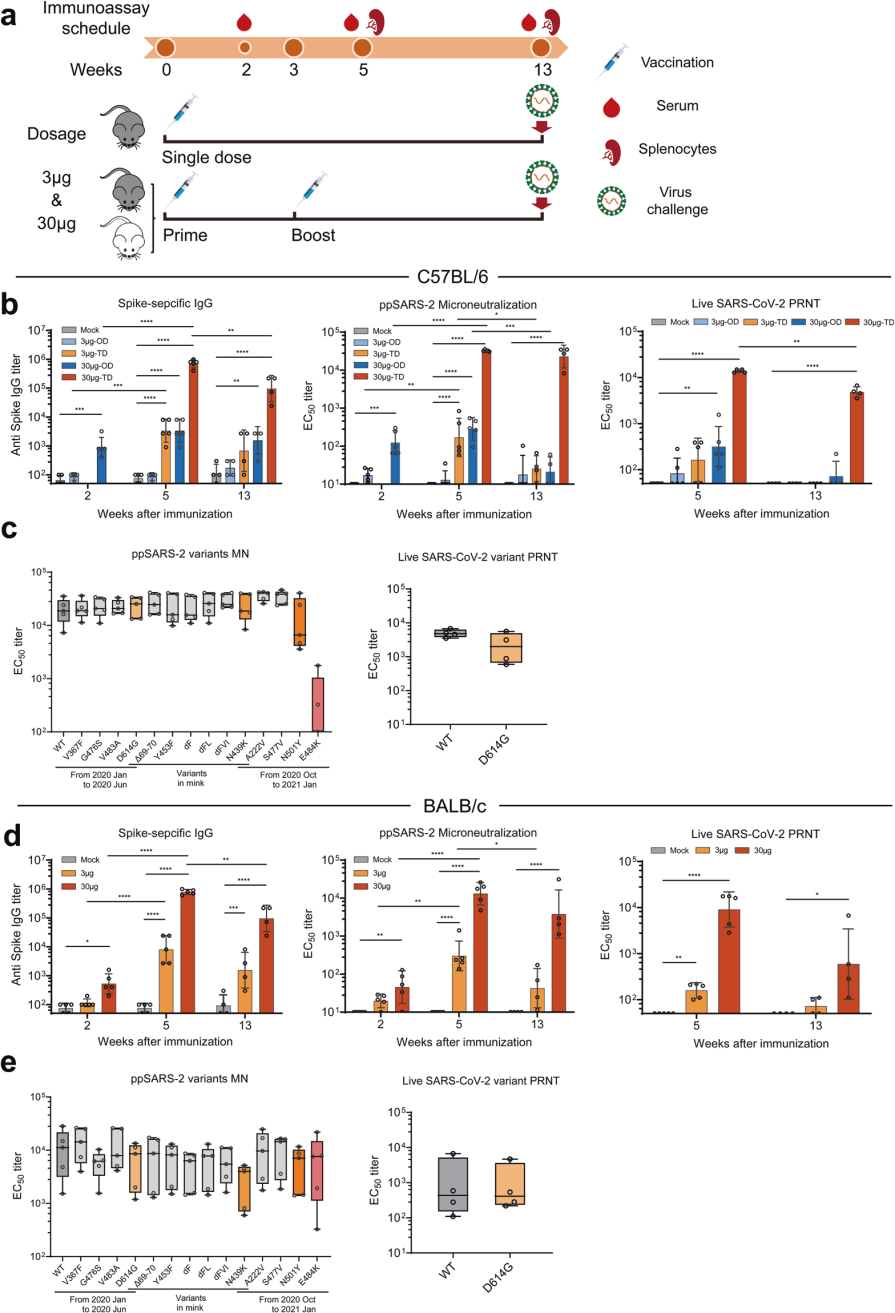
Induction of robust and persistent levels of Abs with broadly neutralizing capacity by SW0123. **a** Two different strains of mice were used to evaluate immunogenicity and protective efficacy of vaccines. C57BL/6 mice received either an one-dose (OD) vaccination with 3 or 30 μg SW0123 (based on mRNA content) in week 0 or two-dose (TD) vaccinations with the same dosage in weeks 0 and 3. BALB/c mice were immunized twice with 3 or 30 μg SW0123 in weeks 0 and 3. Mice in the mock group were administrated with empty vector as control. S protein-specific IgG titers were measured in weeks 2, 5, and 13 using ELISA. (*n* = 5 in weeks 2 and 5; *n* = 4 in week 13) (**b**, **d** left panel). Neutralizing Ab (NAb) titers were measured using a SARS-CoV-2 pseudovirus microneutralization assay (**b**, **d** mid panel) or using a live SARS-CoV-2 (Wuhan/IVDC-HB-01/2019) plaque reduction neutralizaion test (**b**, **d** right panel). NAb titers are shown as EC50 values, calculated by Reed-Muench method. **c**, **e** Serum collected at week 5 from two-dose SW0123-immunized mice (*n* = 5 for each strain) were tested for neutralizing ability against pseudotype SARS-CoV-2 with indicated mutation points in S protein or were tested against live D614G mutant and wide-type strain (Wuhan/IVDC-HB-01/2019) using plaque reduction neutralization test. Two-tailed Mann–Whitney test was used for statistical analysis. *p ≤ 0.05; **p < 0.01; ***p < 0.001; ****p < 0.0001

A dose-escalating evaluation of SW0123 was first performed in C57BL/6 mice, which showed a dose-dependent induction of S protein-specific IgG and IFN-γ-secreting T cells following SW0123 administration (Supplementary Fig. 2A and 2B). The efficacy of SW0123 upon storage up to 4–6 weeks was also evaluated and compaired side-by-side with fresh SW0123. No change of immunogenicity was observed (Supplementary Fig. 2C). Further in-depth evaluations of immunogenicity and protective activity of SW0123 were carried out in two murine strains (Fig. 3a). C57BL/6 mice were immunized i.m. with one dose or two doses of 3 or 30 μg SW0123, and the kinetics of vaccine responses were assessed based on dose and dosing frequency, while BALB/c mice received a two-dose immunization schedule of 3 or 30 μg vaccine. Five days before viral challenge, the immunized animals were sensitized for SARS-CoV-2 infection using adenovirus expressing hACE2 transduction technology to render the mice susceptible to SARS-CoV-2 challenge as described.^11^

Regardless of mouse strain, the mice receiving two doses of SW0123 all produced robust levels of S protein-binding IgG after 5 weeks. The responses were more prominent in the groups receiving the high dose (30 μg) and were maintained throughout the 13-week study (Fig. 3b, d, left). A single immunization of 30 μg SW0123 elicited lower IgG levels than two immunizations of the same dose did, but the response was comparable to that generated by two immunizations of 3 μg vaccine (Fig. 3b, left). Neutralizing capacity of Abs was assessed with two complementary assays: a pseudovirus neutralization assay (PNA) and a live virus plaque reduction neutralization test (PRNT). In line with IgG titers, the highest level of neutralizing Abs was observed in mice receiving two doses of 30 μg SW0123, reaching 21,000 and 12,000 reciprocal EC50 geometric mean titers at week 5 in C57BL/6 mice based on the PNA and PRNT assays, respectively (Fig. 3b, right). Similar response was observed in BALB/c mice (Fig. 3d, right). Moreover, SW0123 induced higher levels of IgG2 than IgG1 (Supplementary Fig. 3), indicating that a Th1-polarized immune response is induced by nucleic acid vaccines as expected.^2,24^

Next, we assessed whether Abs from SW0123-immunized mice were able to neutralize SARS-CoV-2 variants, and confirmed a high neutralizing activity against majority of the pseudotyped SARS-CoV-2 variants tested (Fig. 3c, e, left). However, the capacity to neutralize the E484K variant was dramatically decreased in C57BL/6 mice, but was not found in BALB/c mice. We also compared neutralizing ability of Abs against the wild-type SARS-CoV-2 and the live D614G mutant using the PRNT assay, and found equal levels of neutralization (Fig. 3c, e, right). Taken together, these results suggest that SW0123 is able to provide broad protection against a panel of SARS-CoV-2 variants.

### SW0123 induced strong Th1-biased T cell responses

**Fig. 4:**
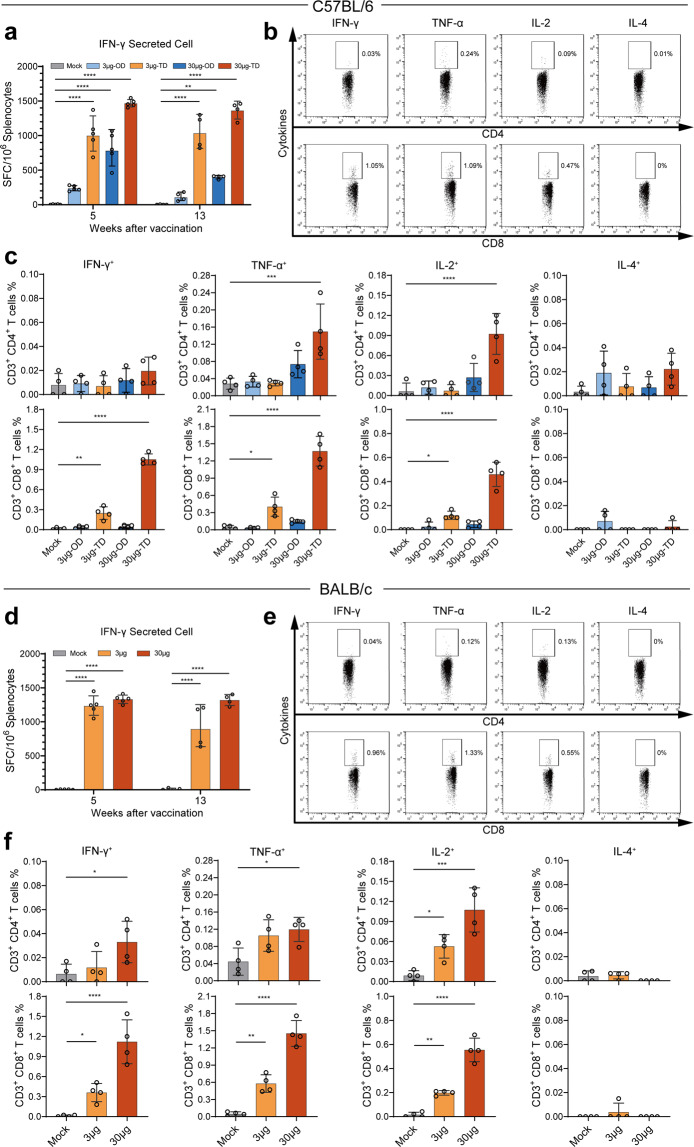
Induction of predominantly Th1-biased T cell responses by SW0123. C57BL/6 mice received either an one-dose (OD) vaccination with 3 or 30 μg SW0123 in week 0 or two-dose (TD) vaccinations with the same dosage in weeks 0 and 3. BALB/c mice were immunized twice with 3 or 30 μg SW0123 in weeks 0 and 3. Mice in the mock group were administrated with empty vector as control. (*n* = 5 in week 5; *n* = 4 in week 13). **a**, **d** Splenocytes were isolated 5 and 13 weeks after the first vaccination. Following stimulation with S protein overlapping peptides for 20 h, IFN-γ-producing T cells were quantified with an ELISpot assay. **b**, **c**, **e**, **f** Splenocytes isolated 13 weeks after first vaccination were stimulated with S protein overlapping peptides for 6 h, and Spike specific cytokine-producing CD4^+^ and CD8^+^ T cells were measured with an intracellular cytokine recall assay. Two-tailed Mann–Whitney test was used for statistical analysis. *p ≤ 0.05; **p < 0.01; ***p < 0.001; ****p < 0.0001

Although the role of T cells in protection against SARS-CoV-2 is not completely clear, there is a need for sufficient CD4^+^ T helper cell responses to induce high quality Ab responses. In addition, CD4^+^ and CD8^+^ T cell responses directed to S protein have been detected in SARS-CoV-2 infected individuals and showed a clear correlation with NAb titers.^25–27^ To assess T cell responses induced by SW0123 in mice, we stimulated splenocytes from vaccinated mice with a pool of S protein-specific peptides, and performed ELISpot assay to quantify the number of S-specific T cells. A robust and Th1-polarized T cell response was elicited upon immunization, demonstrated by increased levels of S-specific IFN-γ-producing T cells (Fig. 4a, d). In addition, T cell responses were maintained for 13 weeks with no significant decline as compared with that measured at week 5, which is in line with the sustained Ab responses (Fig. 3). T cell response at week 13 was further evaluated using an intracellular cytokine staining (ICS) assay. SW0123 induced significantly higher levels of S-specific CD4^+^ and CD8^+^ T cells that predominantly secreted Th1-type cytokines including IFN-γ, IL-2, and TNF-α, but not IL-4, a Th2-type cytokine (Fig. 4b, c, e, f). The results are consistent with the high IgG2/IgG1 ratio observed in vaccinated mice (Supplementary Fig. 3) and thus further confirm that SW0123 vaccination induces a Th1-skewed vaccine response.

### SW0123 induced protection against SARS-CoV-2 infection both in mice and rhesus macaques

**Fig. 5:**
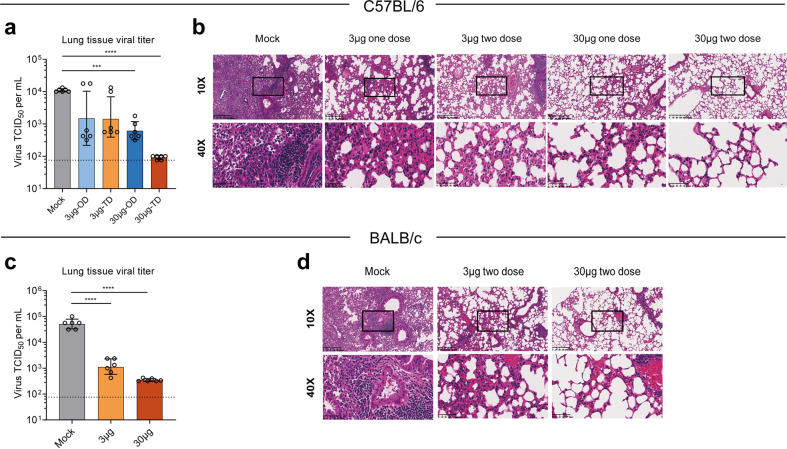
Induction of efficient protection against SARS-CoV-2 in mice by SW0123. C57BL/6 mice received either an one-dose (OD) vaccination with 3 or 30 μg SW0123 in week 0, or two-dose (TD) vaccinations with the same dosage in weeks 0 and 3. BALB/c mice were immunized twice with 3 or 30 μg SW0123 in weeks 0 and 3. Mice in the mock group were administrated with empty vector as control. Mice (*n* = 6 each group) were transduced with rAdV5-expressing hACE2 13 weeks after first dosing, and were challenged intranasally with 5 × 10^5^ TCID50 of SARS-CoV-2 (Wuhan/IVDC-HB-01/2019) 5 days of post-transduction. Lung tissues were harvested 4 days of post-challenge. **a**, **c** Viral titer levels in the lungs. Right lungs were homogenized and determined for viral titers. **b**, **d** Histology of lung sections. Left lungs were sectioned for hematoxylin and eosin (H&E) staining. Two-tailed Mann–Whitney test was used for statistical analysis. ***p < 0.001; ****p < 0.0001

Given the sustained NAb titers and vaccine-specific T cells in vaccinated animals, we next evaluated protective effect of SW0123 in mice 13 weeks post vaccination. A well-established mouse challenge model using adenovirus expressing hACE2 transduction technology to render the mice susceptible to SARS-CoV-2 infection was used in the study.^11^ Mice in the mock control groups receiving administration of LPP empty vector exhibited high viral loads in the lungs 4 days after SARS-CoV-2 exposure, while those in the vaccine groups had significantly decreased viral loads (Fig. 5a, c). The level of protection was most profound in mice vaccinated twice with the high-dose SW0123 as demonstrated by a nearly complete protection with over 100-fold decrease of viral load. Lung histopathology showed that the mock-vaccinated mice had severe rupture of pulmonary alveoli, excessive mucus production, and immune cell infiltration. In contrast, SW0123-immunized mice presented only focal and mild histopathological changes (Fig. 5b, d). This effect was particularly prominent in mice that received two high-dose vaccinations. We also found that C57BL/6 mice receiving a single high-dose vaccination showed significantly decreased virus titer and clearly ameliorated inflammation and tissue damage in the lung (Fig. 5a, b). In addition, a single or two low-dose vaccinations that elicited low-to-medium levels of Abs also showed a protective effect on the lungs (Fig. 5b, d), which implies that SW0123 is unlikely to trigger an antibody-dependent enhancement (ADE) effect even when given at a sub-protective dose.

**Fig. 6:**
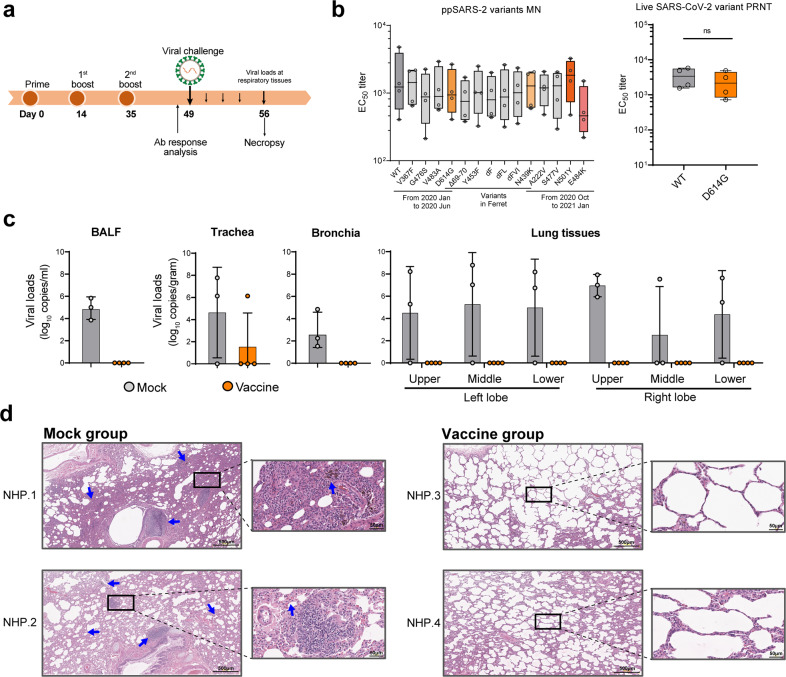
Induction of sufficient protection against SARS-CoV-2 in rhesus macaques by SW0123. **a** Schematic study design. Rhesus macaques received three doses of SW0123, and were intranasally and intratracheally challenged with 1 × 10^6^ PFU of SARS-CoV-2 two weeks after the 2nd boost. Animals in mock group received PBS injection. Samples were collected at the indicated time points. **b** Titers of NAbs for neutralizing ability against pseudotype SARS-CoV-2 with indicated mutation points in S protein or were tested against live D614G mutant and wide-type strain (Wuhan/IVDC-HB-01/2019) using plaque reduction neutralization test. **c** Animals were euthanized and necropsied at 7 dpi. gRNA copies in trachea, bronchia, BAL fluid and lung tissues were determined. **d** H&E staining of lung tissue sections from animals. Blue arrows point to lung pathological lesions. Images from two representative animals in each group are shown. Two-tailed Mann–Whitney test was used for statistical analysis. *p ≤ 0.05; **p < 0.01; ***p < 0.001; ****p < 0.0001

Rhesus macaques (RMs) are naturally susceptible to SARS-CoV-2 infection and are important models for evaluation of COVID-19 vaccines.^28,29^ We therefore evaluated immunogenicity and protective capacity of SW0123 in this animal model. RMs were immunized with three doses of 200 μg SW0123 at a 2-week interval, followed by intranasal and intratracheal inoculation with 1 × 10^6^ PFU of SARS-CoV-2 two weeks after the final immunization (Fig. 6a). Prior to viral challenge, all vaccinated RMs displayed high levels of NAbs with equal neutralizing ability against all mutants in the panel (Fig. 6b). Nasal and throat swab specimens were collected every other day following infection for determination of viral genomic RNA (gRNA) copies. A persistent and high level of viral gRNA was detected in nasal swabs from mock-treated RMs. In contrast, vaccinated animals had consistently reduced levels of viral gRNA beginning on day 3 of post-infection (Supplementary Fig. 4). Further, viral loads in multiple tissues of respiratory system were determined following necropsy. High copies of viral gRNA were present in bronchia and bronchoalveolar lavage fluids (BALF) of mock-treated animals, while in contrast, all the four vaccinated animals showed no detectable viral gRNA in these specimens (Fig. 6c). In line with these findings, no viral loads were detected in the lung tissues of any of the vaccinated animals, while all the animals in the mock group had high viral loads. Further, histological examination showed severe lung damages following SARS-CoV-2 infection in unvaccinated animals, manifested as thickened alveolar septa, low-to-moderate hemorrhage, as well as inflammatory exudates in intraluminal spaces (Fig. 6d). The vaccinated RMs, on the other hand, displayed intact pulmonary alveoli structure with mild focal histopathological changes. Collectively, these results demonstrate that SW0123 conferred highly effective protection against SARS-CoV-2 infection in RMs.

### Safety profiles of SW0123

While the current pandemic situation demands rapid development of effective vaccines, safety is undoubtfully a prerequisite and should not be compromised. Towards that goal, we have systemically assessed the safety profiles of SW0123 in different animal models in compliance with Good Laboratory Practice (GLP) (Supplementary Table 1). No systemic adverse events were observed. Immediate adverse events following vaccination included a transient and rapid increase of body temperature within 24 h and a temporary elevation of serum IL-6 level. Increased numbers of white blood cells, monocytes and neutrophils that returned to normal range within 2–3 days were also observed (data not shown). These symptoms are normal reactions to vaccination as a result of a temporary innate activation. Similar to other vaccines,^30^ local reactions at vaccination site were observed, including swelling, redness and bruising. Long-term safety profile of SW0123 was particularly focused (Supplementary Table 2). Cynomolgus macaques received four doses of vaccination at 2-week intervals followed by a 4-week recovery period. Overall, no obvious body weight change, morbidity or mortality was observed and minor side effects (erythema and/or edema) were limited to the injection site. No significant change in ALT or AST was observed, which was in line with the aforementioned limited accumulation of SW0123 in the liver (Fig. 2c–e). These results collectively indicate a mild reactogenicity of SW0123.

## Discussion

In this study, based on the proprietary core-shell structured LPP-mRNA delivery system, we developed a highly efficacious mRNA vaccine (SW0123) against SARS-CoV-2. As the key component in the vaccine formulation, LPP is endowed with multiple advantages including superior colloidal stability, high encapsulation and delivery efficiency, as well as desirable biodistribution pattern. Different from other mRNA delivery platforms, the mRNA vaccine produced by LPP platform can be stored and transported under 4 °C conditions. Also, the unique combination of size and composition of LPP allowed retention of the vaccine particles at the injection site, thus preventing vaccine particles from triggering organ-specific side effects, for example liver accumulating effect observed for LNP-mRNA formulations shown in this study and also by others.^18,23^ Such biodistribution pattern may lead to activation of liver-resident cells that subsequently cause tissue damage and inflammation, thus contributing to high reactogenicity. An additional advantage of the LPP-mRNA system is their ability to augment TLR7/8 signaling activation in dendritic cells, resulting in secretion of type I interferons.^31^ Suppression of the interferon response has been shown to be an important mechanism of SARS-CoV-2 in the pathogenesis of the viral disease.^32^ Thus, the LPP-mRNA vaccine may be especially potent to induce a strong antiviral response.

Different from the two licensed mRNA vaccines (mRNA-1273 and BNT162b) using mRNA encoding prefusion-stabilized S protein as immunogen,^7,33^ SW0123 is prepared with mRNA encoding the full-length S protein in the natural state. All the three mRNA vaccines have demonstrated robust immunogenicity and strong protective activity. Importantly, the induced vaccine responses were all biased to Th1 phenotype, which was represented by the induction of both Th1-type Abs and T cells. In both mice and NHPs, SW0123 was highly immunogenic to induce Ab and T cell responses, which remained at a high level in vaccinated mice throughout the 13-week study duration, indicating the prospects of long-term protection. A number of recent studies have indicated a rapid decrease of Ab titer in patients infected with SARS-CoV-2.^8,9^ These observations highlight the necessity to develop vaccines that are able to induce sustained immune responses.

The emergence of SARS-CoV-2 variants has raised concerns for causing potential loss of protection from COVID-19 vaccines developed based on early virus isolates. SARS-CoV-2 D614G has become a dominant variant worldwide, with remarkably enhanced infectivity and transmissibility.^20^ The variants such as Y453F, N439K, N501Y, and E484K have shown either enhanced cross-species transmissibility or ability to escape neutralization by NAbs.^21,34,35^ Although some of the mutations occurred in RBD region such as V367F, G476S, V483A, SW0123-elicited Abs were still able to neutralize these variants effectively, which suggested that diversified NAbs and non-RBD region functional Abs can be induced. Of note, although E484K mutation causes escape from convalescent serum and monoclonal Abs,^36^ sera collected from SW0123-immunized BALB/c mice and rhesus macaques still maintained high neutralizing activity against this variant. But interestingly, SW0123-elicited Abs in a C57BL/6 mouse showed a significantly decreased capacity to neutralize this variant. This may be due to the genetically divergent germline antibody repertoires in two different inbred mouse strains.^37^

Using both mouse and rhesus macaque SARS-CoV-2 challenge models, a protective ability of SW0123 was confirmed. In mice, the maximumly efficient protection was achieved through two high-dose vaccinations as expected. Although a single low-dose vaccination elicited low levels of NAbs, a clearly moderate protective effect on the lungs was also observed, which suggested that low-level Abs may to some extent contribute to protection and that other immune parameters may also be involved, which merits further investigations to better understand the immune correlates of protection against SARS-CoV-2. On the other hand, the low-dose vaccination did not generate an ADE-dependent immunopathological effect in mice upon viral challenge, which has become a major concern in COVID-19 vaccine development due to previous evidence of this in the context of MERS and SARS infections.^38,39^ In rhesus macaques, SW0123 demonstrated a nearly complete protection, represented by a rapid and efficient clearance of inoculated virus both in the upper and lower respiratory tract and also no evident lung pathology.

Longitudinal studies of COVID-19 patients with disease severity spanning from asymptomatic/mild to fatal have indicated the role of multiple immune cell subsets, e.g., myeloid derived suppressor cells (MDSCs) and nature killer (NK) cells, in the prediction of disease severity and host protection.^40,41^ In addition, the roles of functional SARS-CoV-2 specific T cells in viral clearance and disease resolution are increasingly acknowledged.^42^ A strong correlation between the level of SARS-CoV-2 specific T cells and NAbs have been revealed in COVID-19 patients.^26^ Interestingly, a robust virus-specific memory T cell response was also detected in antibody-seronegative patients, and convalescent individuals with a history of asymptomatic infection.^43^ All these suggested that both NAbs and functional T cells are likely required to confer an efficient protection. To this end, mRNA vaccine is a preferential vaccine platform as the two licensed COVID-19 mRNA vaccines have demonstrated strong ability in eliciting both NAbs and Th1-type T cells in humans. Collectively, this study demonstrates strong immunogenicity, protective ability and high safety profiles of SW0123, which makes this vaccine a promising candidate for future clinical evaluation. SW0123 is now under evaluation in a Phase I clinical trial in China (Chinese Clinical Trial Registry, CTR20210542).

## Materials and methods

Additional methods are available in supplementary Materials and Methods.

### Biosafety and ethics

All work with live SARS-CoV-2 in mouse model was performed in Biosafety Level 3 (BSL-3) containment laboratories at the National Institute for Viral Disease Control and Prevention, Chinese Centers for Disease Control and Prevention (China CDC). BALB/c and C57BL/6 mice (Charles River Laboratories) were housed in National Institute for Viral Disease Control and Prevention or National Institute of Occupational Health and Poison Control. Chinese rhesus macaques (4 years old, male) were provided and housed in Kunming National High-level Biosafety Primate Research Center, China. All work with live SARS-CoV-2 in NHP model was performed in ABSL-4 facility. All animal studies were conducted under the ethical regulations and were approved by local ethical committees.

### Immunization and sampling

C57BL/6 and BALB/c mice (8-week-old) were randomly allocated to the indicated groups. Mice received a single-dose or a prime-boost immunization of different doses of SW0123. Vaccines were injected into each thigh at a volume of 50 μL using insulin syringe (BD Biosciences). Blood was collected from the orbital sinus under anaesthesia, followed by centrifugation and serum collection. For the evaluation of T cell response, spleens were excised and single cell suspension of splenocytes were prepared for immune assays. In nonhuman primate experiment, rhesus macaques (4 years old) were i.m. immunized with 200 μg SW0123 at day 0, day 14, and day 33 in a volume of 100 μL. Blood was drawn into BD Vacutainer® Plus Plastic Serum Tubes for serum collection.

### Enzyme-linked immunosorbent assays

Recombinant SARS-CoV-2 Spike protein (50 ng, Sino Biological) diluted in carbonate buffer (0.1 M, pH 9.6) were coated into 96-well EIA/RIA plates (Coning) overnight at 4 °C. The plates were then washed with PBS-T (0.05% Tween-20) and were blocked with 10% goat serum in PBS for 2 h at 37 °C. Then, serum samples serially diluted in PBST containing 2% goat serum were added and incubated for 2 h at 37 °C. After washing, total IgG was evaluated using HRP-conjugated goat anti-mouse IgG Ab (1:10,000) or HRP-conjugated goat anti-NHP IgG Ab (1:50,000) for 1 h. In mice experiments, IgG subclasses were evaluated using biotinylated anti-mouse IgG2a mAb (clone: MG2a, Mabtech), biotinylated anti-mouse IgG1 mAb (clone: MG1, Mabtech), biotinylated anti-mouse IgG2c mAb (clone: MTG2c, Mabtech) for 1 h and further analyzed with Streptavidin-HRP (1:1000, Mabtech). TMB substrate (Solarbio) was used for development and the absorbance was read at 450 nm using SPECTROsta Nano (BMG) microplate reader. Endpoint titers were calculated as the dilution that exceed 2.1-folded value of the background.

### Pseudovirus neutralization assay

Pseudovirus neutralization assay was performed as previously reported.^44^ In brief, gene sequence of SARS-CoV-2 (GISAID, No. EPI_ISL_402119) S protein was codon optimized for expression in human cells. SARS-CoV-2 variants containing specific single amino acid mutation in S protein were generated by genetic modification (GenScript) according to reported sequence obtained from GISAID.^10,21,34,45^ Plasmids expressing S protein and plasmids encoding a defective HIV-1 genome (pNL4-3. Luc. R-E-) expressing a luciferase reporter were co-transfected into HEK 293T cells using X-treme GENE HP DNA Transfection Reagent (Roche). Cell culture was refreshed 6 h after transfection, and cell suspensions enriched with the pseudotype virus were harvested after 48 h and were stored at −70 °C. Pseudovirus-containing supernatants were incubated with or without serial dilution of heat-inactivated (HI) serum for 1 h at 37 °C. Subsequently, the mixture was added to 60–70% confluent Huh7.5 cells seeded in 96-well plates and was incubated for 12 h. Cells cultured alone or cultured with only SARS-CoV-2 pseudoviruses were run in parallel. Media was refreshed once with DMEM (2% FBS) and the incubation was continued for 48 h at 37 °C. Following this, cells were lysed, and luciferase signal was measured using Bright-Glo firefly luciferase kit (Promega). Percentage of neutralization was calculated and EC_50_ titers were determined.

### Intracellular cytokine staining

A total of 2 × 10^6^ splenocytes (100 μL) were seeded per well into 96-well plates and incubated with S protein overlapping peptide (5 μg/mL) for 6 h at 37 °C. Monensin (Thermo Scientific) was added 1 h after the start of culture. Unstimulated cells were used as control. After stimulation, cytokine production of memory T cells were evaluated by surface and intracellular cytokine staining using Fixation/Permeabilization Solution kit (BD Biosciences) as previously reported.^46^ Fluorochrome-conjugated antibodies used for surface staining (Thermo Scientific) include: anti-CD3e PerCP-eFluor 710 (eBio500A2), anti-CD4 AF488 (GK1.5) and anti-CD8a PE (53-6.7), anti-IFNγ APC (XMG1.2), anti-TNF APC (MP6-XT22), anti-IL-2 APC (JES6-5H4), and anti-IL-4 APC (11B11). Splenocytes were analyzed on Accuri C6 (BD Biosciences). One hundred thousand events were collected per sample. Frequency of S protein-specific T cells was detemined by flow cytometric analysis.

### SARS-CoV-2 challenge experiment

In the viral challenge experiments using BALB/c and C57BL/6 mice, hACE2 transduced SARS-CoV-2 challenge mouse model was generated as described previously.^11^ In brief, mice were first lightly anesthetized with isoflurane and transduced intranasally with 2.5 × 10^8^ PFU of Ad5-hACE2 in 40 μL of saline buffer 5 days prior to challenge. Five days post transduction, mice were intranasally infected with 5 × 10^5^ TCID50 of SARS-CoV-2 (Wuhan/IVDC-HB-01/2019) in a total volume of 50 μL. Mice were monitored at a daily basis for mortality. Four days of post-challenge, mice were euthanized, and necropsy was performed. In viral challenge experiments using rhesus macaques, animals were anesthetized and then intranasally and intratracheally challenged with 1 × 10^6^ PFU of SARS-CoV-2 two weeks after the 2nd boost immunization. At 1, 3, 5, 7 dpi, nasal swabs and throat swabs specimens were collected for determination of viral genomic and subgenomic RNA copies by qPCR as previously reported.^47^ At 7 dpi, animals were anesthetized and necropsied. Trachea, bronchia, bronchoalveolar lavage fluid (BALF), and lung tissues from upper, middle, and lower lobes of left or right lungs were collected.

### Statistical analysis

Unpaired non-parametric Mann–Whitney test was used to compare values between two groups. One-way ANOVA was used to compare values in over two groups. All statistical analyses were conducted with GraphPad Prism 7.0. A *p* value less than 0.05 was considered as statistically significant. *p ≤ 0.05; **p < 0.01; ***p < 0.001; ****p < 0.0001.

## Supplementary information

Supplementary Materials for A core-shell structured COVID-19 mRNA vaccine with favorable biodistribution pattern and promising immunity

## Data Availability

The data sets used for the current study are available from the corresponding author upon reasonable request.
