# Supplementary material for: A core-shell structured COVID-19 mRNA vaccine with favorable biodistribution pattern and promising immunity
Source: Signal Transduct Target Ther. 2021 May 31;6:213. doi: 10.1038/s41392-021-00634-z (PMC8165147; doi:10.1038/s41392-021-00634-z)
Supplement: Supplementary file 1 — Supplementary Materials for A core-shell structured COVID-19 mRNA vaccine with favorable biodistribution pattern and promising immunity [file 41392_2021_634_MOESM1_ESM.docx]

Supplementary Materials for

A core-shell structured COVID-19 mRNA vaccine with favorable biodistribution pattern and promising immunity

Ren Yang, Yao Deng, Baoying Huang, Lei Huang, Ang Lin, Yuhua Li, Wenling Wang , Jingjing Liu, Shuaiyao Lu, Zhenzhen Zhan, Yufei Wang, Ruhan A , Wen Wang, Peihua Niu, Li Zhao, Shiqiang Li, Xiaopin Ma, Luyao Zhang, Yujian Zhang, Weiguo Yao, Xing-Jie Liang, Jincun Zhao, Zhongmin Liu ,Xiaozhong Peng^#^, Hangwen Li^#^, Wenjie Tan^#^

**Correspondence to:** Wenjie Tan, tanwj@ivdc.chinacdc.cn; Hangwen Li, lihangwen@stemirna.com; Xiaozhong Peng, pengxiaozhong@pumc.edu.cn

**This PDF file includes:**

Materials and Methods

Figures. S1 to S4

Tables S1 to S2

Materials and Methods

mRNA synthesis

SARS-CoV-2 Spike protein gene was codon optimized and inserted into in-vitro transcription vector, which flanked with the 5’ and 3’ untranslated regions and a 120nt poly-A tail. T7 RNA polymerase-mediated transcription was employed to synthesize the mRNA from tail-PCR produced DNA template, which 50% UTP was substituted with 1-methylpseudo UTP. After this, in vitro transcription products were incubated at 37°C for 8 hours, followed by treatment with DNAse. RNA was capped using Vaccinia Capping Enzyme and purified using magnetic dynabeads, and then treated with Antarctic Phosphatase at 37℃ for 30 minutes to remove residual 5’-triphosphates. Capped mRNA was then purified by colum chromatography and dissolved in 25 mM sodium acetate (pH 5.2). Quality of mRNA was analyzed by agarose gel electrophoresis and stored at -70℃.

Preparation of LPP nanoparticles.

LPP nanoparticles were prepared by a two-step method. Briefly, a cationic compound (SW-01) was dissolved in 25 mM sodium acetate (pH5.2) and diluted with RNAase-free water. mRNA/SW-01 complexes were prepared by mixing the mRNA solution（pH4, 10 mM citrate buffer）and SW-01 solution at volume ratio of 5:1 (mRNA: SW-01) and stand for 30 min at room temperature. After that, lipids were dissolved in ethanol at weight ratios of 49: 49: 2 (ionizable lipid: 1,2-dioleoyl-sn-glycero-3-phosphoethanolamine (DOPE): PEG-lipid). The lipid mixture was combined with mRNA/SW-01 complexes at a ratio of 3:1 (aqueous: ethanol) using a microfluidic mixer (Inano D, Micro&Nano Technology Inc, China). Formulations were dialyzed against PBS (pH 7.4) in dialysis cassettes for at least 12 hr. Formulations were concentrated using Amicon ultra centrifugal filters (EMD Millipore), passed through a 0.22-mm filter, and stored at 4℃.

Gel electrophoresis and immunoblotting

mRNA- or sham-transfected cells were lysed for protein extraction. Protein samples were denaturated by mixing with 5 × SDS loading buffer and were subsequently separated on 4–10% polyacrylamide TGX gel (Bio-Rad) for 1h 20 min at 120 V. After this, proteins were transferred to Nitrocellulose membrane under semi-dry condition: 23 V for 25min. The membrane was blocked by incubating with 5% skim milk overnight at 4℃. S protein was detected using polyclonal rabbit anti-S protein Ab (1:2000, Sino biological) and β-actin was detected using mouse Anti-β-actin monoclonal antibody(1:2000, Abcam) for 1 h at room temperature (RT), followed by incubation with goat anti-rabbit or anti-mouse IgG-HRP Ab (1:3000, Sigma) for 1 h. After all incubations, the membrane was washed with PBST for 15 min and proteins were visualized using immobilon western chemilum HRP substrate (Millipore) under ECL imaging system.

Preparation of LNP

Lipid components were dissolved in ethanol at molar ratios of 50:10:38.5:1.5 (ionizable lipid: DSPC: cholesterol: PEG-lipid). The lipid mixture was combined with a 10-mM citrate buffer (pH 4.0) containing mRNA at a volume ratio of 3:1 (mRNA: lipid) and total flow rate of 12 mL/min using a NanoAssembler system (Inano D, Micro&Nano Technology Inc, China). Formulations were dialyzed in 10-kDa membrane dialysis cassettes against phosphate-buffered saline (PBS, pH 7.4) for at least 24 h, followed by concentration using 100-kDa Amicon ultracentrifugal filters, filtration through a 0.22-mm filter.

mRNA concentration and encapsulation efficiency of LPP-mRNA

The mRNA content in LPP-mRNA was quantified by Quant-iT™ RiboGreen™ RNA Reagent (Thermo Scientific). SW0123 was diluted first with nuclease-free water and 1 × TE buffer (10 mM Tris-HCl, 0.1 mM EDTA, pH 8.0), followed by mixture with an equal volume of 2% Triton X-100 and incubation at RT for 1 hour to release the encapsulated mRNA-SW-01 core. 100 μL of each sample was then transferred to the wells of a 96-well plate, to which 100 μL of 200 × diluted RiboGreen™ RNA Reagent was added. The plate was shaken and incubated at RT for 10 min. After that, the fluorescence of the plate was read by a Bio-Tek Synergy I plate reader (BioTek). A series of mRNA-SW-01 calibration standards were also prepared corresponding to 0.1–2.0 μg/mL mRNA. The calibration standards were treated with Triton X-100 and RiboGreen™ RNA Reagent in parallel with the samples. A calibration curve of fluorescence and mRNA concentration was plotted by linear regression, from which the mRNA concentrations of the samples were calculated. Encapsulation efficiency of SW0123 was defined as the percentage of encapsulated mRNA out of total mRNA amount in the test sample. To evaluate the stability of SW0123 following storage at 4℃, mRNA concentration and encapsulation efficiency were determined at different time point after SW0123 was produced.

Biodistribution of LNP-luciferase-mRNA and LPP-luciferase-mRNA

BALB/c mice were i.m. injected with 10 µg of luciferase-mRNA delivered by LPP or LNP. 6 hours later, animals were intraperitoneally injected with 30 mg of D-luciferin firefly (Maokang Biotechnology) and bioluminescence was measured in a Xenogen IVIS-200 imaging system. Upon necropsy, major organs including liver, spleen, lung, kidney, injection site draining lymph node, heart and brain were harvested and homogenized. Level of luciferase expression was determined by SuperLight™ Luciferase Reporter Gene Assay Kit (Bioassay).

Biodistribution of SW0123

BALB/c mice were i.m. injected with SW0123 at a single dose of 1.5mg/kg. A variety of specimens including whole blood, heart, liver, spleen, lung, kidney, stomach, small intestine, brain, fat, muscle tissue at injection site, and mediastinal lymph node were collected at 2, 6, 24, 72, 168 and 240 hour following vaccination (n=6 mice each time point). Tissue samples were weighed and stored at RNA Later^TM^. Blood samples were stored at TRIzol™ LS Reagent. Specimens were analyzed for mRNA concentration by real-time qPCR using probes specific for the mRNA component of SW0123 and calculated using an established standard curve. Area under curve (AUC) was calculated, which represents accumulative distribution of SW0123 in each specimen over 360-hour duration post vaccination.

In-vitro mRNA transfection and signal detection

Immobilized cell lines were transfected with mRNA component of SW0123, luciferase-mRNA or eGFP-mRNA using Lipofectamine MessengerMAX (Thermo Scientific) or LPP system. For the experiments where mRNA component of SW0123 was evaluated, HEK293T and DC2.4 cells were transfected with mRNA using Lipofectamine according to the instruction. Briefly, 1 μg of mRNA was mixed with 3 μL of Lipofectamine reagent first and then added to cell cultures for 48 or 96 hours. For the experiments where eGFP-mRNA was transfected, 1 x 10^5^ DC2.4 cells were incubated with 0.5 μg of eGFP-mRNA packaged in LPP or lipofectamine (Thermo Scientific) for 24 h, and eGFP expression was visualized using an Eclipse DMi8 fluorescent microscope (Leica). Frequency of eGFP-expressing cells was determined using flow cytometry (BD FACSCanto II, Becton Dickinson).

Plaque reduction neutralization test (PRNT)

Live SARS-CoV-2 strain Wuhan/IVDC-HB-01/2019 and live D614G mutant was produced in Vero cells (ATCC CCL-81). 80 plaque forming unit (PFU) of viruses were incubated with serial dilution of heat-inactivated serum in DMEM medium (Hyclone) for 1 h at 37 °C. The virus–serum mixture (300 μL) was added to a 90% confluent monolayer of Vero cells in 12-well plate and incubated for 1 h at 37 °C with intermittent shaking. Then, cells were overlaid with 1 ml of DMEM containing 1.2% Avicel RC-581 (FMC Biopolymer), 2% FBS and 1% penicillin/streptomycin (Gibco), followed by a 3-day incubation at 37 °C. The medium was then aspirated and cells were fixed and stained with 0.1% crystal violet (Sigma) in 25% methanol. Wells were rinsed with deionized water to visualize plaques. Neutralization titers were calculated to effective dilute per milliliter and EC50 titers were determined by Reed-Muench method. Values below the limit of detection are reported as half of the limit of detection.

Enzyme linked immunospot (ELISpot) assay

MAIPSWU 96-well plates (Millipore) were coated with 100 ng of IFN-γ capturing Ab (BD bioscience) per well at 4℃ overnights, and then blocked by RPMI 1640 complete medium. Single cell suspension of splenocytes were diluted in RPMI 1640 complete medium at a concentration of 5 × 10^6^ cells per ml. 5 × 10^5^ cells (100 μL) cell were added to plates and incubated in the presence of overlapping S protein peptides (4μg/ml) for 20 hours. After washing, plates were incubated with biotinylated secondary Ab (1:250, BD bioscience) for 2 hours at RT, followed by incubation with Streptavidin-HRP (1:100). Spots were developed with 3-Amino-9-ethylcarbazole (AEC) substrate for 10 minutes and were couted by Bioreader 4000-PRO-X (BIOSYS).

Viral titer determination

In mouse challenge experiments, mice were anesthetized and lungs were excised. Right lungs were removed and placed in DMEM, and then homogenized using a tissue homogenizer. Lung homogenates were centrifuged and supernatant was collected. Serial dilutions of the supernatant were added to 90% confluent Vero cells in 96-well plate in 8 replicates. After 96 hours, 50% tissue culture infective dose (TCID50) were determind by Reed-Muench method. In NHP challenge experiments, tissue samples were homogenized using a tissue homogenizer following collecteion at necropsy. Viral genomic and subgenomic RNA copies in tissues were measured as previously reported^1^. Primers and probes specific for *NP* gene were used and synthesized : forward: 5’-GGGGAACTTCTCCTGCTAGAAT-3’; reverse: 5’-CAGACATTTTGCTCTCAAGCTG-3’; probe: 5’-FAM-TTGCTGCTGCTTGACAGATT-TAMRA-3’).

Histology

Following necropsy, mice and rhesus macaques were anesthetized and lungs were excised. Lung tissues were rinsed in PBS twice, fixed in 4% Paraformaldehyde (PFA), and then paraffin embedded. Tissue blocks were sectioned at 5 μm and then stained with hematoxylin and eosin (HE) for histological examination. All images were taken using NIKON CI-S and DS-U3 camera. Images were analyzed by 2 independent pathologists that are blinded to the study design.

1. Lu, S. *et al.* Comparison of nonhuman primates identified the suitable model for COVID-19. *Signal Transduct Target Ther*. **5**, 157, (2020).


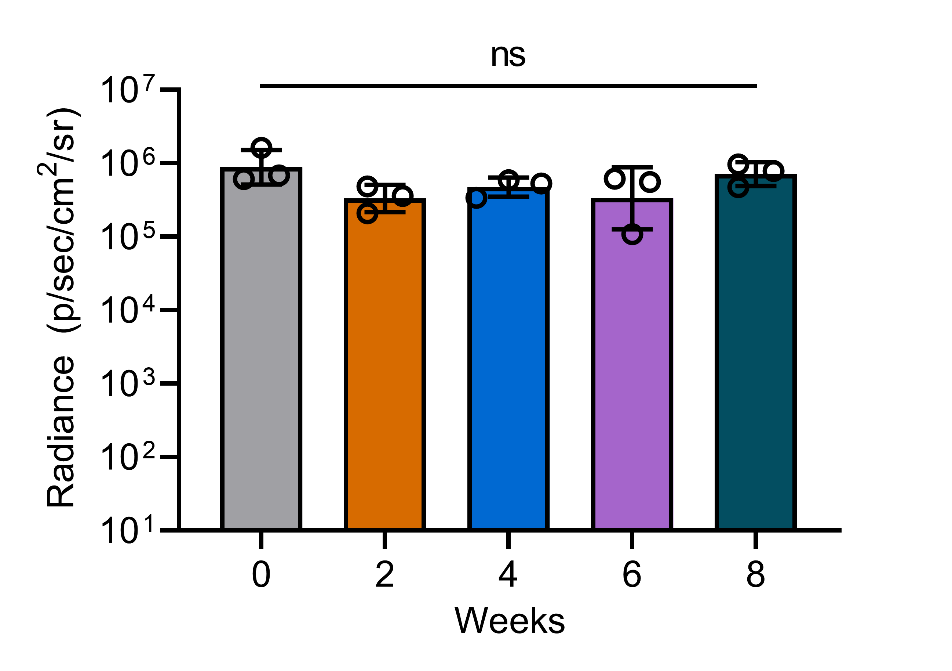


Figure. S1. Translation efficiency of LPP-mRNA during storage.

BALB/c mice were treated intramuscularly with 5 µg LPP-luciferase-mRNA that had been stored at 4℃ for different time, and fluorescent signals at the injection sites were measured 6 hours later.


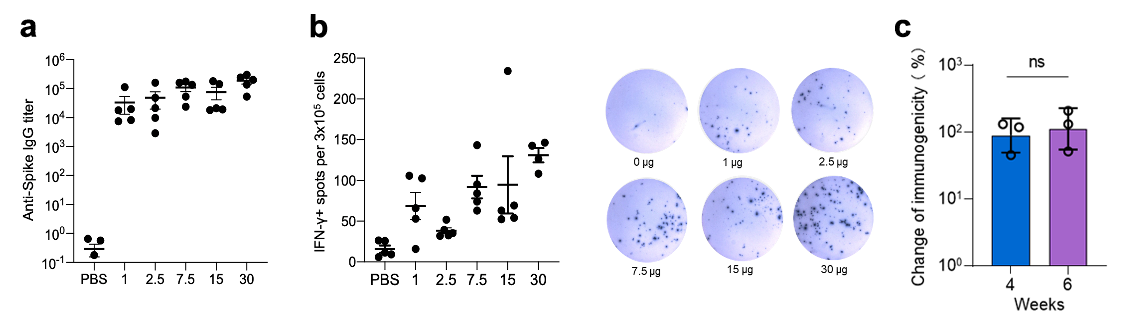


Figure. S2. A dose-escalating evaluation of SW0123 and changes in immunogenicity during storage.

(A). C57BL/6 mice were immunized with escalating doses of SW0123 at a 2-week interval. S protein-specific IgG was measured at 14 days after boost immunization. (B). Splenocytes were isolated upon necropsy and were stimulated with Spike protein for 20 hours. Frequency of IFN-γ-producing T cells were quantified using ELISpot assay. (C). Changes in immunogenicity from SW0123 with different storage time. C57BL/6 mice were immunized with SW0123 at a 2-week interval. S protein-specific antibody was measured 14 days after boost immunization and was compared with the antibody titer elicited by fresh SW0123.


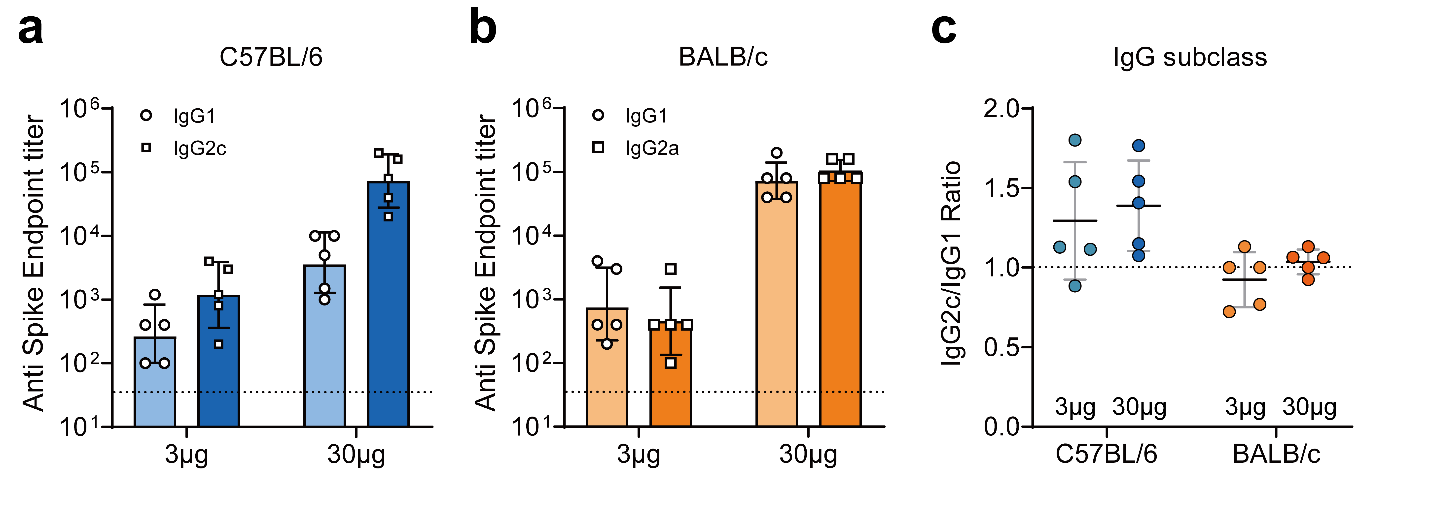


Figure. S3. Levels of IgG subclasses induced by SW0123 vaccine.

Serum samples were collected 5 weeks after the 2^nd^ dosing of SW0123. Titers of S protein-specific IgG subclasses (IgG1 and IgG2) were evaluated. Ratio of IgG subclasses is shown.


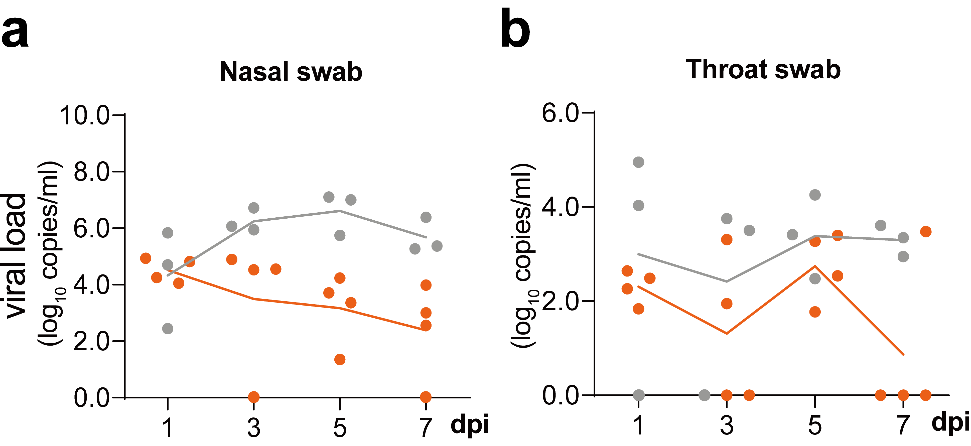


Figure. S4. Viral load in nasal or throat swabs in rhesus macaques infected with SARS-CoV-2.

Mock control (n=3) and vaccinated (n=4) rhesus macaques were intranasally and intratracheally challenged with 1×10^6^ PFU of SARS-CoV-2. Viral genomic RNA (gRNA) copies in nasal swab and throat swab specimens were measured at indicated time points after infection.

Table S1. Pharmaco-toxicological evaluation of SW0123 in animal models

| **Type of experiment** | | **Animal model** | **Number / Sex** | **Administration route** | **Doses** | **Results** |
| --- | --- | --- | --- | --- | --- | --- |
| Neuroethological reaction | | SD rat | 35 (M) / 35 (F) | i.m | 1 | NOAEL: 0.2mg/kg |
| Respiratory function | | SD rat | 30 (M) / 30 (F) | i.m | 1 | NOAEL: 0.2mg/kg |
| Dose-dependent toxicity | Single dose | SD rat | 25 (M) / 25 (F) | i.m | 1 | MTD: 0.8mg/kg |
|  |  | CM | 5 (M) / 5 (F) | i.m | 1 | NOAEL: 0.2mg/kg; MTD: 0.4mg/kg |
|  | Multiple doses | SD rat | 150 (M) / 150 (F) | i.m | 1 dose/2 weeks, 4 doses | LOAEL: 0.02mg/kg |
|  |  | CM | 35 (M) / 35 (F) | i.m | 1 dose/2 weeks, 4 doses |  |
| Local sites toxicity | hemolytic test | Rabbit | N.A | in vitro test | 1 | Negative |
|  | Local site test | Rabbit | 9 (M+F) | i.m | 3 doses/week | Positive reaction at injection site |
|  | ASA test | Guinea pig | 18 (M) / 18 (F) | i.m | 5 | Negative |
| Genotoxicity test | Ames test | N.A | N.A | in vitro test | N.A | Negative |
|  | Micronucleus + Comet assay | SD rat | 35 (M) / 35 (F) | i.m | 1 dsoe/day, 4 consective days | Negative |
| **Abbreviation:** SD: Sprague-Dawley; CM: Cynomolgus macaque; NOAEL: No observed adverse effect Level; LOAEL: Low observed adverse effect level; MTD: Maximum tolerated dose; ASA: active systemic anaphylaxis test | | | | | | |

Table S2. Changes of serum biochemical parameters in cynomolgus macaques receiving repeated administration of SW0123

|  | | Day 7 | | | | | Day 44 | | | | |
| --- | --- | --- | --- | --- | --- | --- | --- | --- | --- | --- | --- |
|  |  | Control | LPP-eGFP  0.2mg/kg | SW0123-0.02mg/kg | SW0123-0.05g/kg | SW0123-0.2mg/kg | Control | LPP-eGFP  0.2mg/kg | SW0123-0.02mg/kg | SW0123-0.05g/kg | SW0123-0.2mg/kg |
| ALT (U/L) | Mean | 40.8 | 46.8 | 60.2 | 62.2 | 39.4 | 52.8 | 33.8 | 67.2 | 38.0 | 34.8 |
|  | SD | 9.7 | 33.2 | 25.7 | 41.8 | 18.7 | 12.7 | 14.1 | 43.8 | 10.9 | 9.9 |
|  | %Diff | N/A | 14.7 | 47.5 | 52.5 | -3.4 | N/A | -36.0 | 27.3 | -28.0 | -34.1 |
| AST(U/L) | Mean | 45.4 | 53.8 | 47.2 | 53.4 | 40.2 | 54.4 | 40.0 | 54.2 | 44.8 | 46.2 |
|  | SD | 10.9 | 31.2 | 10.6 | 15.6 | 10.3 | 12.8 | 4.9 | 20.3 | 7.9 | 13.6 |
|  | %Diff | N/A | 18.5 | 4.0 | 17.6 | -11.5 | N/A | -26.5 | -0.4 | -17.6 | -15.1 |
| ALP (U/L) | Mean | 410.2 | 389.4 | 335.6 | 346.6 | 346.8 | 396.6 | 417.4 | 408.6 | 438.2 | 382.0 |
|  | SD | 103.6 | 98.0 | 113.7 | 47.4 | 71.1 | 73.3 | 64.3 | 136.5 | 86.8 | 95.8 |
|  | %Diff | N/A | -5.1 | -18.2 | -15.5 | -15.5 | N/A | 5.2 | 3.0 | 10.5 | -3.7 |
| Total Protein  (g/L) | Mean | 73.2 | 67.0 | 72.2 | 67.6 | 67.6 | 73.8 | 68.2 | 72.4 | 68.6 | 68.8 |
|  | SD | 3.6 | 1.9 | 5.9 | 6.4 | 6.1 | 5.2 | 2.8 | 4.6 | 3.9 | 4.0 |
|  | %Diff | N/A | -8.5 | -1.4 | -7.7 | -7.7 | N/A | -7.6 | -1.9 | -7.0 | -6.8 |
| Albumin (g/L) | Mean | 40.4 | 33.4** | 36.4 | 35.8 | 34.0** | 39.2 | 33.2 | 35.0 | 33.2 | 33.0 |
|  | SD | 3.0 | 2.9 | 3.4 | 2.4 | 3.1 | 4.2 | 1.5 | 0.7 | 2.7 | 3.3 |
|  | %Diff | N/A | -17.3 | -9.9 | -11.4 | -15.8 | N/A | -15.3 | -10.7 | -15.3 | -15.8 |
| Globulin  (g/L) | Mean | 32.8 | 33.60 | 35.8 | 31.80 | 33.60 | 34.6 | 35.00 | 37.4 | 35.40 | 35.8 |
|  | SD | 1.64 | 3.29 | 3.63 | 4.09 | 4.62 | 2.41 | 2.92 | 4.04 | 1.82 | 3.56 |
|  | %Diff | N/A | 2.4 | 9.1 | -3.0 | 2.4 | N/A | 1.2 | 8.1 | 2.3 | 3.5 |
| Albumin/Globulin ratio | Mean | 1.23 | 1.01* | 1.02 | 1.13 | 1.02 | 1.14 | 0.95 | 0.94* | 0.94* | 0.93* |
|  | SD | 0.11 | 0.18 | 0.10 | 0.08 | 0.16 | 0.13 | 0.10 | 0.09 | 0.07 | 0.16 |
|  | %Diff | N/A | -18.4 | -17.2 | -8.1 | -16.9 | N/A | -15.9 | -16.9 | -17.4 | -18.0 |
| Lipase  (U/L) | Mean | 15.602 | 17.664 | 23.100 | 16.846 | 20.646 | 1.98 | 4.70 | 3.40 | 23.770 | - ^a^ |
|  | SD | 6.062 | 3.335 | 8.538 | 3.750 | 15.759 | 0.25 | 1.66 | - ^a^ | - ^a^ | - ^a^ |
|  | %Diff | N/A | 13.2 | 48.1 | 8.0 | 32.3 | N/A | 138.0 | 72.2 | -13.5 | - ^a^ |
| Lactate dehydrogenase  (U/L) | Mean | 612.4 | 975.6 | 574.8 | 818.2 | 560.2 | 539.4 | 641.6 | 599.4 | 720.0 | 698.0 |
|  | SD | 369.8 | 544.1 | 111.1 | 153.8 | 85.0 | 159.6 | 127.0 | 165.0 | 180.8 | 111.7 |
|  | %Diff | N/A | 59.3 | -6.1 | 33.6 | -8.5 | N/A | 18.9 | 11.1 | 33.5 | 29.4 |

Cynomolgus macaques were injected with SW0123 and control groups on day 1, day 15, day 29 and day 43 at two-week intervals and followed with a four-week recovery period. During dosing period, blood samples were taken on day 7 and day 44 to measure biochemical parameters. mRNA encoding irrelevant antigen protein GFP was packaged in LPP (LPP-eGFP) as negative control.

Statistically significant differences are shown in bold. AST, aspartate aminotransferase; ALT, alanine aminotransferase; ALP, alkaline phosphatase.

*, p < 0.05; **, p < 0.01; ***, p < 0.001, compared with control.

- ^a^, statistics were done.

N/A, not applicable.
